# Different TLR4 expression and microglia/macrophage activation induced by hemorrhage in the rat spinal cord after compressive injury

**DOI:** 10.1186/1742-2094-10-112

**Published:** 2013-09-10

**Authors:** Yu-Kai Zhang, Jin-Tao Liu, Zheng-Wu Peng, Hong Fan, An-Hui Yao, Peng Cheng, Ling Liu, Gong Ju, Fang Kuang

**Affiliations:** 1 Institute of Neurosciences, Fourth Military Medical University, Xi’an 710032, China; 2 Department of Neurosurgery, The 208th Hospital of PLA, Changchun 130011, China; 3 School of Stomatology, Fourth Military Medical University, Xi’an 710032, China; 4 Department of Psychosomatic Medicine, Xijing Hospital, Fourth Military Medical University, Xi’an 710032, China

**Keywords:** Hemorrhage, Toll-like receptor 4, Microglia/macrophage, Spinal cord injury, Blood-spinal cord barrier, Rat

## Abstract

**Background:**

Hemorrhage is a direct consequence of traumatic injury to the central nervous system and may cause innate immune reactions including cerebral Toll-like receptor (TLR) 4 upregulation which usually leads to poor outcome in the traumatic brain injury. In spinal cord injury (SCI), however, how hemorrhage induces innate immune reaction in spinal parenchyma remains unknown. The present study aimed to see whether blood component and/or other factor(s) induce TLR4 and microglia/macrophages involved innate immune reactions in the rat spinal cord after traumatic injury.

**Methods:**

Using the compressive SCI model of the rat, hemorrhage in the spinal cord was identified by hematoxylin-eosin staining. Microglia/macrophage activation, TLR4 expression, and cell apoptosis were investigated by immunohistochemistry. Nuclear factor (NF)-κB p50 level of the two segments of the cord was detected by western blotting assay. With carbon powder injection, blood origination of the hematoma was explored. The blood-spinal cord barrier (BSCB) states of the lesion site and the hematoma were compared with immunohistochemistry and tannic acid-ferric chloride staining.

**Results:**

Histological observation found blood accumulated in the center of compression lesion site (epicenter) and in the hematoma approximately 1.5 cm away from the epicenter. TLR4 expression, microglia//macrophage activation, and subsequent apoptosis in the area of far-away hematoma were late and weak in comparison to that in epicenter. In addition, TLR4 positive microglia/macrophages appeared to be phagocytotic in the far-away hematoma more obviously than that in the epicenter. Injected carbon powder indicated that accumulated blood of the far-away hematoma originated from the bleeding of the lesion epicenter, and the BSCB around the hematoma was not compromised in the early phase. Accordingly, at 3 days post injury, NF-κB p50 was upregulated based on the similar levels of blood component hemoglobin, and cell apoptosis was obvious in the epicenter but not in the far-away hematoma.

**Conclusion:**

These data suggest that besides blood component, BSCB compromise and the extent of tissue injury contribute more to TLR4 and microglia/macrophage responses to the spinal cord hemorrhage. Therefore, the innate immune environment is a necessary consideration for the SCI therapy targeting TLR4 and microglia/macrophages.

## Background

Intraparenchymal hemorrhage is a direct consequence of traumatic injury to the central nervous system (CNS), and the blood entered in the parenchyma may cause a serial responses including innate immune reactivity and becomes neurotoxic [1]. How the innate immune system of the CNS responds to the hemorrhage is critical for understanding the mechanisms of the secondary injury and making the therapeutic strategies.

As an important class of innate immune receptors, Toll-like receptors (TLRs) expressed on CNS glia and neurons may recognize endogenous ligands as well as invaded pathogen and participate both in development and in responses associated with CNS injury [2]. TLRs have been implicated in both infectious and non-infectious CNS pathology and appear to play important roles in both tissue surveying and repair, and have been considered as therapeutic targets in CNS inflammation and infection [3]. Among the members of TLR family, TLR2 and TLR4 recognize damage molecular pattern (DAMP) as well as pathogen molecular pattern (PAMP) to mediate frontier defense in the CNS [4]. DAMP comprises cell debris, heat shock proteins released from dead neurons, fibrinogen, and other molecules that may damage the healthy tissue further [5]. Therefore, TLR2 and TLR4 are critical for injury induced immune reaction of the CNS. There has been a growing body of evidence that brain trauma induces TLR2 and/or TLR4 upregulation [6-8]. Accumulated studies showed that TLR4 was upregulated in intracerebral hemorrhage (ICH) [9,10], and TLR4 mediated inflammation may lead to poor outcome in these individuals [11]. Moreover, it has been documented that heme, the main component of erythrocyte, activated TLR4-mediated inflammatory injury in ICH [12].

Spinal cord injury (SCI) as a severe traumatic injury to the CNS usually leads to paralysis and sensation disability, and there is still no effective therapy [13]. SCI occurs commonly in modern society because of traffic accidents, sports accidents, and falling from high buildings. The spinal cord is part of the CNS, connecting the brain and the peripheral nervous system, however, whether or how TLR4 responds to hemorrhage after traumatic SCI still lacks report.

In our previous study with the rat SCI model, we documented that TLR4 mRNA was upregulated in the injured spinal cord, and early blockade of downstream signal of TLRs may reduce inflammatory reaction and be helpful to neuroprotection [14], but whether TLR4 is upregulated by hemorrhage, even by blood component in the spinal cord remains unknown.

Pathologically, SCI was divided into primary injury and secondary injury phases [15,16]. Hemorrhage, bone fracture, tissue damage, and cell death directly resulted from violent forces belong to primary injury, which initiates serial lesion called secondary injury. The secondary injury phase is the only part that could be interposed to rescue or protect the cord because the primary injury is unexpected. Primary injury-induced inflammation, ischemia, and hypoxia are considered the pivotal factors that determine the outcome of SCI, but the mechanisms of the secondary injury are still not fully understood [17]. TLR signals tailor the innate immune response [18], which is vital for the CNS restoration of tissue repair and hemoestasis [19]. Therefore, to reveal TLR4 response to hemorrhage in SCI would be helpful to further understand the role of hemorrhage in the secondary spinal cord injury.

In our previous study on SCI models, we found that hematoma distal to the center of the lesion site often occurred in the compressive injury to the rat spinal cord. Preliminary observation indicated that microglia behaved differently in these hemorrhagic foci. We hypothesized that hemorrhage induced the innate immune reaction in the spinal cord, and the lesion center and the peripheral hematoma may produce TLR4 and microglia/macrophage responses differently due to the innate immune environment other than the blood component. In this study, microglia/macrophage activation and TLR4 response to hemorrhage in the distal hematoma as well as in the epicenter of the lesion were investigated. Distinct pattern of microglia/macrophage activation and TLR4 activities were seen in the epicenter and distal hematoma. Meanwhile, since hemorrhage resulting from a vascular event also means blood-spinal cord barrier (BSCB) breakdown which exposes the CNS to circulation and causes neurotoxic effect [19], the present study also explored the origination of the blood in the distal hematoma and the BSCB states in the hemorrhagic areas. The data indicated that different situations of BSCB compromise might contribute more to those different innate immune responses. These findings may give new insight to spinal cord hemorrhage as well as the innate immune reaction of SCI.

## Methods

### Animals and experimental protocol

Male Sprague–Dawley rats (approximately 220–250 g), provided by the Experimental Animal Center of the Fourth Military Medical University, were maintained with temperature (approximately 22-25°C) and light (12-h light/dark cycle) control, and had free access to water and food. All the experiments involving animals were approved by the Animal Care Committee of the Fourth Military Medical University.

A spinal cord compressive model was made, and animals were randomly divided into various groups. The numbers of animals assigned to those experiments are shown in Table 1. At 6 h, 3 days, and 14 days post injury, animals were sacrificed by intra-cardiac perfusion with 4% paraformaldehyde, and the spinal cord were removed and sectioned for histological observation. Another batch of animals was sacrificed by decapitation and their spinal cords were taken for western blotting assay. To explore the source of blood forming distal hematoma, carbon powder was injected into the compressive epicenter before compression, and hemisection in dorsal cord were made in six other rats. In order to explore the BSCB compromise, six rats were subjected to tannic acid-ferric chloride staining and immunohistochemistry for rat IgG.

**Table 1:** Animal assignment for all experiments and time points

	**6 h**	**3 days**	**14 days**
H-E and immunohistochemistry	3	4	3
Western blotting assay		4	
Carbon powder injection	3		
Hemi-transection		3	
Tannic acid-Ferric chloride staining	3		

### Compressive spinal cord injury model

**Figure 1:**
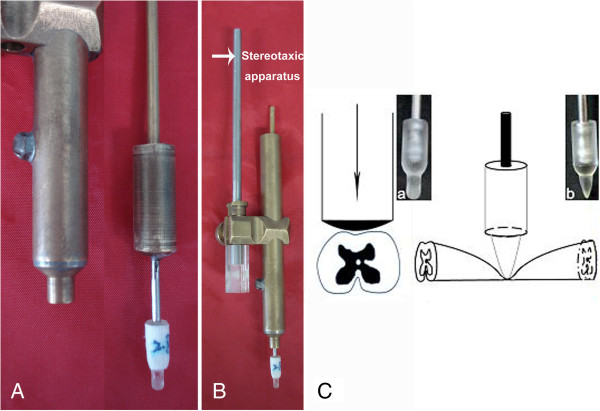
**The device for the compressive spinal cord injury model of the rat. (A)** The metal tube and the rod with a plastic plate attached onto the tip. **(B)** The rod is held by the tube which could be connected with the stereotaxic apparatus. **(C)** Schematic diagram of compressive injury to the spinal cord by the plastic plate attached to the rod tip, **(a)** front view and **(b)** side view.

Compressive injury model was performed as previously reported [20]. A 20 g metal rod was used to make the compress injury (Figure 1). The tip of the rod was covered with a plastic plate which is approximately 2.6 to 2.9 mm wide (depends on the diameter of the cord) and 0.5 mm thick. The rod was held by a metal tube which controls the position and the depth of the rod via connection with the sterotaxic apparatus but does not press the rod anytime. Before the compression injury, the rat was anesthetized with 1% sodium pentobarbital (50 mg/kg, i.p.), a 30–40 mm dorsal midline incision was made, then the spinal cord was exposed by a bilateral laminectomy of T8 vertebra (corresponding to T9 segment of the spinal cord), with the dura intact. Then the vertebral column was fixed with stabilizing forceps and the rod was placed onto the spinal cord, with the plastic plate perpendicular to the longitudinal axis of spinal cord, and the tip attached the dura surface. After that, the metal tube was moved down vertically at a speed of 0.5 mm/min with the sterotaxic apparatus to produce compression onto the spinal cord by the weight of the rod. After 5 min, the plastic plate was lowered to the bottom of the vertebral canal to achieve a full compression of the cord. The rod was remained at this position for 1 min, followed by a slow withdraw within 1 min. The wound was closed by suturing muscles and skin over the vertebral column. The rats were kept in cages with soft bedding, and manual evacuation of urinary bladder was performed twice daily.

### Histological observation

Rats were sacrificed at 6 h, 3 days, or 14 days post injury by an overdose of sodium pentobarbital (100 mg/kg) and perfused intra-cardially with 100 mL of warm normal saline followed by 400 mL 4% cold paraformaldehyde in phosphate buffer (pH 7.4). After perfusion, a 2-cm-long spinal cord segment, with the injured site in the middle, was removed and put into 25% sucrose in phosphate buffer at 4°C until it sank to the bottom of the container (at least 12 h). Then serial 20 μm frozen sagittal sections were cut with a cryostat and mounted on slides in eight sets for hematoxylin and eosin (H-E) staining and immunohistochemistry.

For H-E staining, sections were rinsed in distilled water and were stained in hematoxylin solution for 5 min. After washing with running tap water for 5 min, the sections were differentiated in 1% acid-alcohol for 30 s and were washed again with tap water for 1 min. Then the sections were put into Eosin for 30 s, and were dehydrated through 70%, 80%, 90%, and 100% alcohol for 2 min each. After two changes of xylene, sections were covered with xylene-based mounting medium. Then the stained sections were observed to figure out the lesion site and hematoma.

For immunohistochemistry, sections were rinsed with 0.01 M phosphate buffer saline (PBS) and then blocked with 1% bovine serum albumin (Sigma) in PBS containing 0.5% Triton X-100 for 30 min at room temperature. Then the sections were incubated with primary antibody overnight and fluorescence conjugated second antibody for 2 h. To see the serum protein extravasation in the injured spinal cord, rat immunoglobulin G (IgG) was detected by immunohistochemistry. Briefly, spinal sections were directly incubated with Alexa Fluor 488 goat anti-rat IgG for 2 h. Antibodies against IBa-1 (1:500, rabbit polyclonal, Wako, Tokyo, Osaka, Japan), ED1 (1:400, mouse monoclonal, Serotec, Raleigh, NC, USA), TLR4 (1: 200, mouse monoclonal, Abcam, Cambridge, MA, USA), reactive endothelial cell antigen (RECA) (1:500, mouse monoclonal, Abcam, Cambridge, MA, USA) were used. Fluorescence conjugated secondary antibodies were purchased from Molecular Probes, Oregon, USA. Omission of the primary antibody served as the negative control. The sections were observed under an Olympus FV1000 laser scanning confocal microscope.

### Western blotting assay

At 3 days post compression SCI, animals were deeply anesthetized and sacrificed by decapitation (*n* = 4). Spinal cord tissues of 3 cm, with epicenter of the injury in the middle, were removed rapidly. Then the spinal cord was divided into three parts equally as rostral, central, and caudal segments, 1 cm of each. All the spinal cord segments were stored in liquid nitrogen and then processed for extraction of protein. Briefly, tissue samples were homogenized with 0.5 mL of ice-cold lysis buffer (20 mM Tris–HCl, pH 7.5, 1 mM EDTA, 5 mM MgCl_2_, 1 mM DTT, 20 μg/mL aprotinin, 1 mM PMSF, and 2 mM sodium orthovanadate). The homogenates were centrifuged at 13,000 rpm for 10 min at 4°C and supernatant were removed. The protein concentration was determined using Bradford method, a detergent-compatible protein assay with a bovine serum albumin as standard. Samples were boiled at 100°C for 10 min and then were electrophoresed on 10-15% SDS-PAGE and transferred onto a nitrocellulose membrane (Millipore, Bedford, MA, USA). The filter membranes were blocked with 5% BSA for 1.5 h at room temperature and incubated with the primary antibody (NF-κB p50, 1:5,000, Epitomics, CA, USA; phospho-IκB, 1:10,000, Epitomics, CA, USA; Homeglobin-alpha, 1:2,000, Epitomics, CA, USA, β-actin, 1:5,000, Cwbiotec, Beijing, China) for approximately 16 to 24 h at 4°C. The membrane was then washed with TBST buffer and incubated with the secondary antibody conjugated with horseradish peroxidase (1:5,000; Cwbiotec, Beijing, China) for 1 h at room temperature and visualized in ECL solution. The density of specific bands was measured with Image J (NIH, USA) software.

Statistical analysis of the density of the specific bands was made with software SPSS 16.0. Density of band NF-κB p50 and phosphor-IκB were compared with that of Hemoglobin-alpha, respectively, then the ratio of NF-κB p50/hemoglobin-alpha, phosphor-IκB/ hemoglobin-alpha of lesion segments, and far-away hematoma segments were analyzed by paired T test (segments of the same spinal cord was paired, *n* = 4); *P* value at 0.05 was considered significant.

### Origination of blood in distal hematoma

In order to find out the source of the blood that formed hematoma distal to the lesion site, carbon powder injection experiment was designed. Immediately after the compressive injury was completed, 1 μL PBS containing 0.1 g/mL fine carbon powder was injected slowly with a microsyringe into the lesion site, at a depth of approximately 1 mm. After injection, the tip of syringe was kept for 2 min and was withdrawn carefully. The whole process was completed within 10 min.

In the case that bleeding from the lesion may expand to the distal area, transection between the lesion site and the distal area could be a barrier to impede formation of distal hematoma. To prove this hypothesis, dorsal hemisection was made in another group of animal immediately after compression. At sites approximately 0.3 to 0.4 mm rostral and caudal to lesion center, dorsal hemisection was made with a sharp blade at a depth of approximately 1 mm.

All these animals were killed by intra-cardiac perfusion with 4% paraformaldehyde, and the spinal cord was sectioned for histological analyses.

### Blood vessel integrity

BSCB may determine the innate immune reaction. Here in this study, tannic acid-ferric chloride staining plus reactive endothelial cell antigen (RECA) immunohistochemistry were used to show vascular integrity. Firstly, RECA immunolabeling was performed in the spinal section at 6 h post injury, to show the existence of blood vessels in both hemorrhage foci, since RECA is biomarker of endothelial cells. Another batch of animals was killed at 6 h post injury by perfusion with tannic acid and ferric chloride to cover the endothelial cells of the blood vessel. The spinal sections from these animals will be immunolabeled by RECA later. The broken down blood vessels may not be well perfused with tannic acid-ferric chloride, so the RECA immunoreactivity would be positive; otherwise, the endothelial cells of the intact blood vessels would not be labeled with RECA.

Animals were anesthetized with an overdose of sodium pentobarbital and were perfused through intracardially with saline (37°C) followed by 4% paraformaldehyde, 2% tannic acid, and 0.5% glutaraldehyde in 0.01 M PBS (37°C). Then 250 mL ferric chloride solution (3%, 37°C) was perfused immediately and spinal cord was removed promptly after the perfusion.

## Results

### Histological profile of hemorrhage

**Figure 2:**
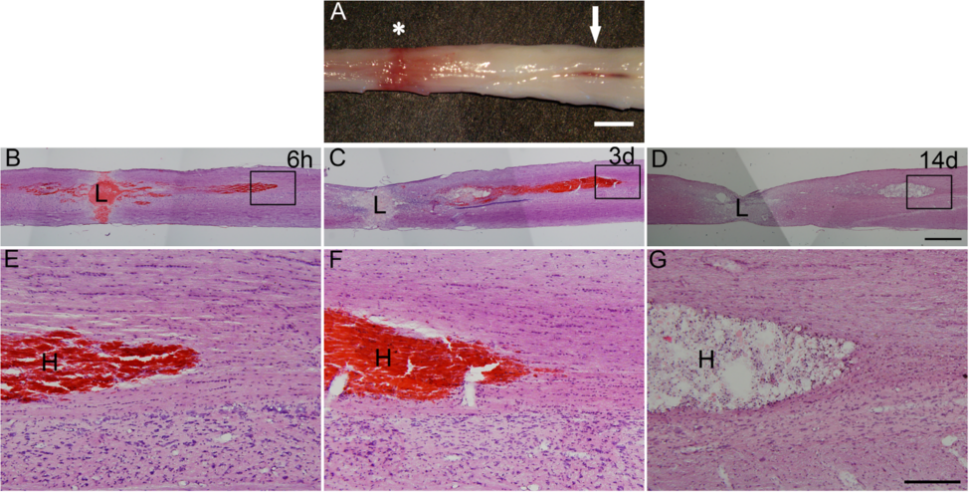
**Compressive spinal cord injury caused distal hematoma far away from the lesion site. (A)** A segment of rat spinal cord taken out immediately after compression. Aster shows the compressive point, and the arrow shows the distal hematoma which could be seen grossly. **(B-D)** H-E staining of the spinal cord sections indicated blood in the compressive lesion center and far-away hematoma which forms spindle-shaped foci at 6 h, 3 days, and 14 d post injury, respectively. **(E-G)** Higher magnification of the boxes in the pictures above accordingly. Note that cavity formed from the hematoma at 14 days post injury. Bar = 1 mm (A-D), 200 μm (E-G).

As shown by H-E staining, hemorrhage in the spinal cord formed two distinct foci, the compressive lesion area and the far-away hematoma. The hematoma usually appeared in the caudal, dorsal part of the cord, approximately 1.5 cm away from the epicenter of the injury, which could be even seen grossly (Figure 2A). Distal hematoma was spindle shaped with clear limitation to spinal cord parenchyma. At 6 h post injury, the far-away hematoma formed, while it developed continuously to a larger hematoma till 3 days post injury. Later at 14 days post injury, the spindle-shaped hematoma became a cavity (Figure 2B-G).

**Figure 3:**
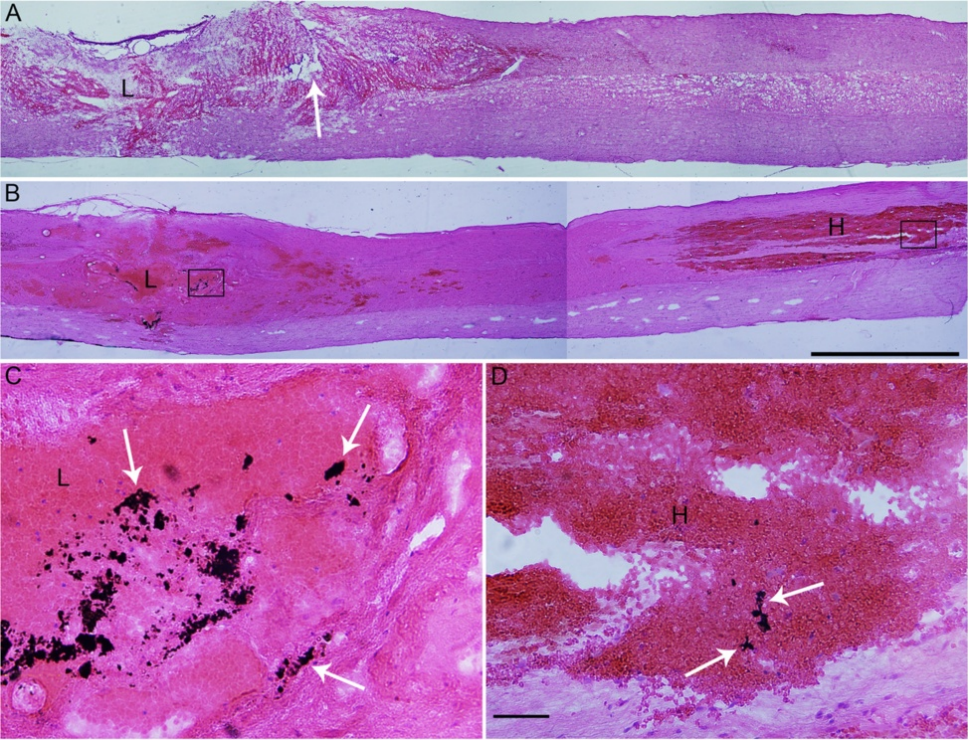
**Blood origination of the far-away hematoma. (A)** H-E staining pictures show that there is no hematoma far away from lesion site in the spinal cord with semi-transection (arrow) aside the lesion. **(B)** Distal hematoma contained carbon powder injected to the compression center immediately after injury, indicating that blood originated from the lesion site. **(C)** and **(D)** are higher amplified pictures of the boxes located in the lesion site (indicated by letter L) and distal hematoma (indicated by letter H) in (B), showing carbon powders (arrows) inside the hemorrhagic foci. Bar = 2 mm in (A) and (B). Bar = 50 μm in (C) and (D).

Carbon powder injected into the epicenter of the injury indicated that the far-away hematoma may originate from the lesion site, because in the far-away hematoma, carbon powder which was injected to the lesion site immediately after the compression was found 6 h post injury (Figure 3B-D). Moreover, in the compression injured spinal cord with hemi-transection in the dorsal part at rostral and caudal sides, there was no far-away hematoma formed even at 3 days post compression (Figure 3A).

### TLR4 expression and microglia/macrophage activation

Immunohistochemistry showed that TLR4 immunoreactivity and activated microglia/macrophage could be seen in both lesion site and far-away hematoma, but differently in time points and distribution.

At 6 h post injury, the immunolabeling of TLR4 and IBa-1 was similar in the lesion site and the hematoma. Some of the IBa-1 positive cells appeared bigger than those in the hematoma, and TLR4 immunolabeling could be found but only in several cells in the epicenter (data not shown).

**Figure 4:**
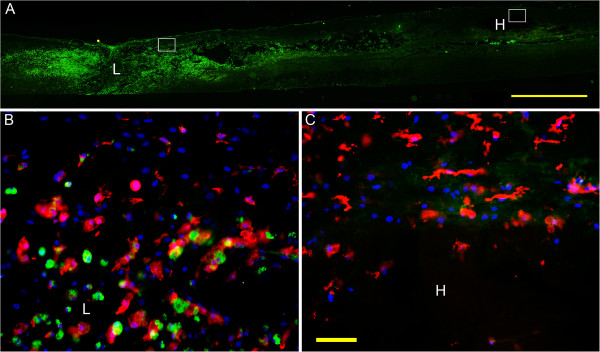
**Microglia/macrophage activation in the spinal area adjacent to the lesion site but not around the distal hematoma at 3 days post injury. (A)** Montage picture of longitudinal spinal cord section for IBa-1 immunolabeling, showing lesion site (indicated by letter L) and hematoma (indicated by letter H) at 3 days post injury. **(B)** Amplified picture of the box adjacent to the lesion site, showing many cells are labeled with both IBa-1 (red), the marker of microglia, and ED-1(green), the marker of activated microglia. **(C)** Amplified picture of the box adjacent to the far-away hematoma in which only IBa-1 positive cells with thin processes could be seen. Bar = 2 mm in (A). Bar = 50 μm in (B) and (C).

At 3 days post injury, a large number of microglia/macrophage appeared in the epicenter, and most of them were activated, indicating by ED1 immunoreactivity and the shape of the cells (Figure 4B). While in the area adjacent to the hematoma far away from the lesion site, most of the IBa-1 positive cells are not ED1 labeled, and their cell body remained small and the processes were thin (Figure 4C).

**Figure 5:**
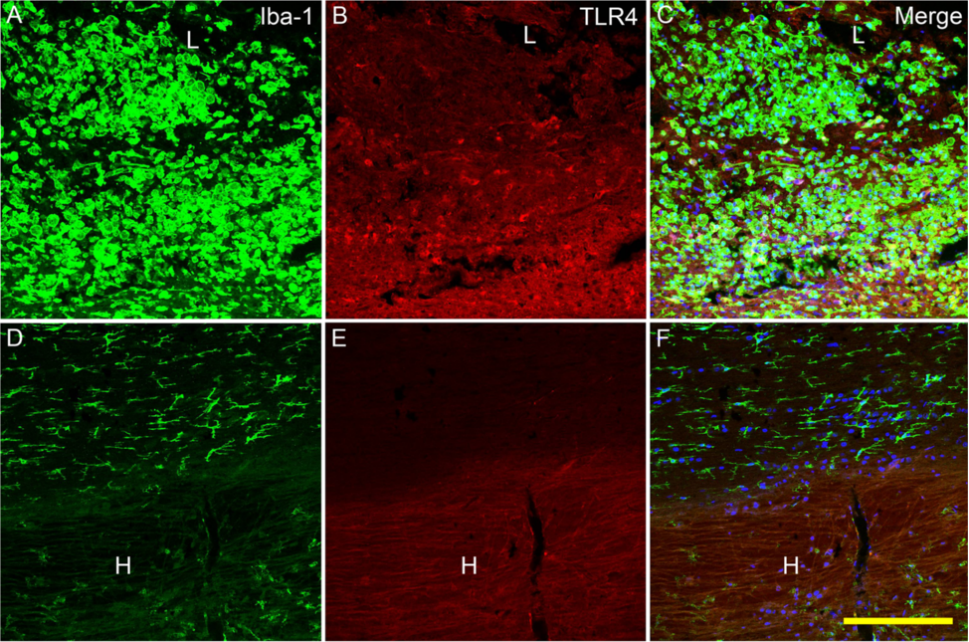
**Immunofluorescent labeling for TLR4 and IBa-1 in the lesion site and the hematoma at 3 days post injury.** In the lesion site **(A-C)**, there were abundant IBa-1 positive cells, quite part of which express TLR4, showed by confocal microscopy. Most of the microglia/macrophages were round with short or blunt processes. To the contrast, in the distal hematoma and the area adjacent to it **(D-F)**, there were few TLR4 positive cells, and IBa-1 labeled cells were small with thin processes. TLR4 immunoreactivity: red; IBa-1 immunoreactivity: green; Hoechest 33342: blue. Bar = 200 μm.

Coming to the TLR4 immunoreactivity, similar profile as microglia/macrophage activity was found. Namely, at 3 days post injury, TLR4 immunolabeling were seen in the center of the compressive lesion, and most of the labeling was co-located with round microglia/macrophage, indicating by confocal microscopy. At the same time point, little TLR4 could be seen in the area of hematoma. Apart from the area around, there were few IBa-1 positive cells inside the hematoma. The distribution of IBa-1 immunoreactivity was clearly limited according to the border of the hematoma (Figure 5).

**Figure 6:**
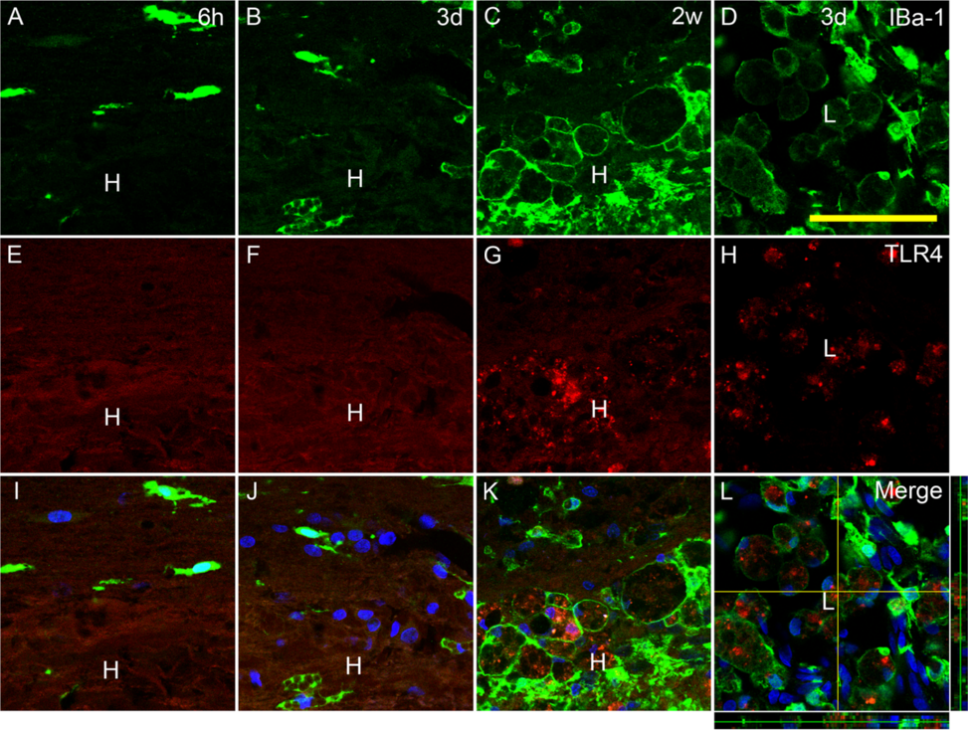
**Representative pictures of immunofluorescent labeling for TLR4 and IBa-1 in the far-away hematoma at 6 h, 3 days, and 14 days post injury.** Note that IBa-1 positive cells are small and scattered in the area adjacent to the hematoma at 6 h **(A, E, I)** and appear in the hematoma at 3 days post injury **(B, F, J)**. At 14 days post injury **(C, G, K)**, TLR4 immunoreactive product is seen in the area of the hematoma, and abundant IBa-1 positive cells are large and phagocytotic and with TLR4 immunoreactivity inside the cytoplasm. All of these observation is similar to the lesion site at 3 days post injury **(D, H, L)**, and the stack of scanning pictures of confocal microscopy confirmed that TLR4 immunoreactive product was located in the cytoplasma of IBa-1 positive cells (L). TLR4 immunoreactivity: red; IBa-1 immunoreactivity: green; Hoechest 33342, blue. L, lesion site; H, hematoma. Bar = 50 μm.

TLR4 immunoreactivity was increased in the hematoma far away from the epicenter of the lesion at 7 days (not shown) and 14 days, which is nearly parallel to the activation of microglia/macrophage. At 14 days, strong TLR4 labeling was seen within the hematoma but not in the adjacent area (Figure 6C, G, K). This profile of TLR4 immunolabeling and microglia/macrophage was similar to that of epicenter at 3 days post injury. Notably, it was clearly shown by confocal microscopy that TLR4 immunoreactivity was located inside the IBa-1 positive cells which were extremely activated as their soma became large and round like huge bubbles (Figure 6D, H, L).

### Cell apoptosis around hemorrhage

**Figure 7:**
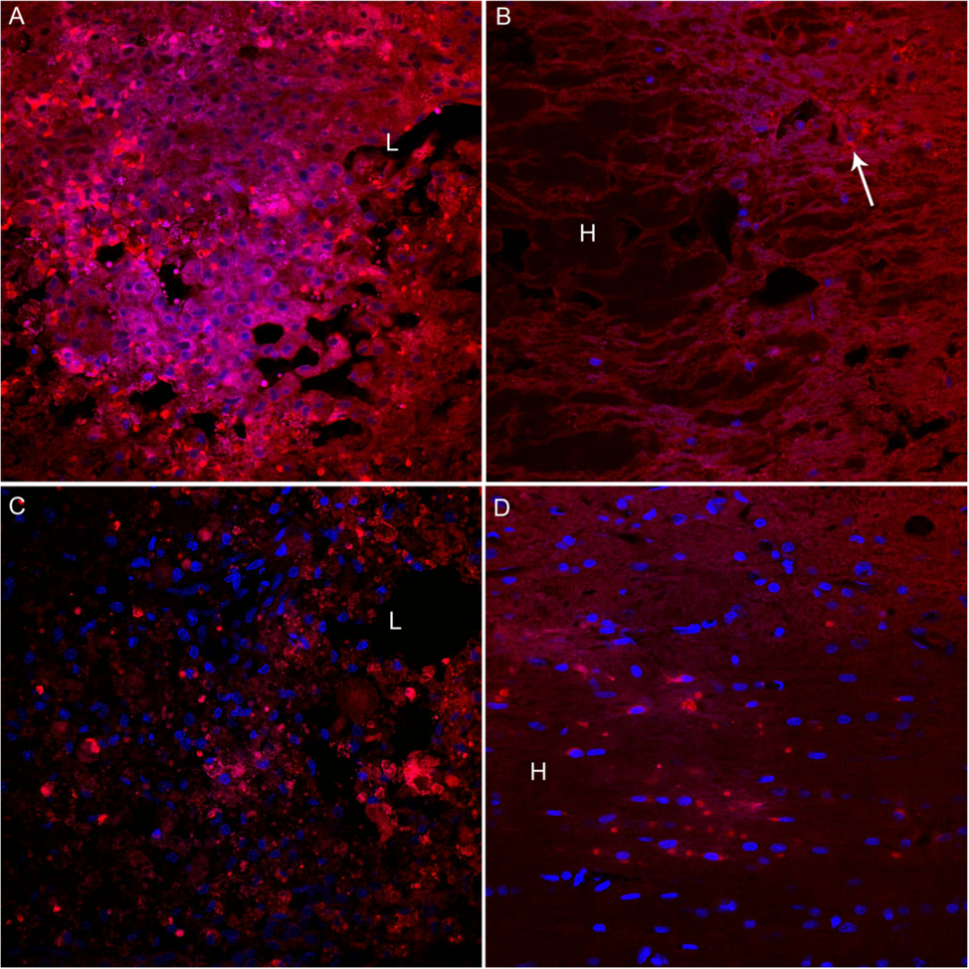
**Immunofluorescent labeling for caspase-3 at 3 days and 14 days post injury.** Representative microscopic photos show numbers of caspase-3 immunoreactive cells inside the lesion area at 3 days post injury **(A)**, while only few cells are caspase-3 positive (arrow) in the area of or adjacent to hematoma **(B)**. At 14 days post injury, there are casepase-3 positive cells in the lesion site but decreased in number **(C)**; while in the area adjacent to the hematoma, caspase-3 immunoreactive cells are markedly increased **(D)**, compared to that at 3 days. TLR4 immunoreactivity, red; IBa-1 immunoreactivity, green; Hoechest 33342, blue. L, lesion site; H, hematoma. Bar = 50 μm.

With immunofluorescent labeling, one can see abundant capase-3 positive cells in the lesion site at 3 days, but very few cells in the far-away hematoma at the same time point. However, the number of capase-3 immunoreactive cells markedly increased in the area around hematoma at 14 days post injury (Figure 7).

### NF-κB upregulated more in lesion site

**Figure 8:**
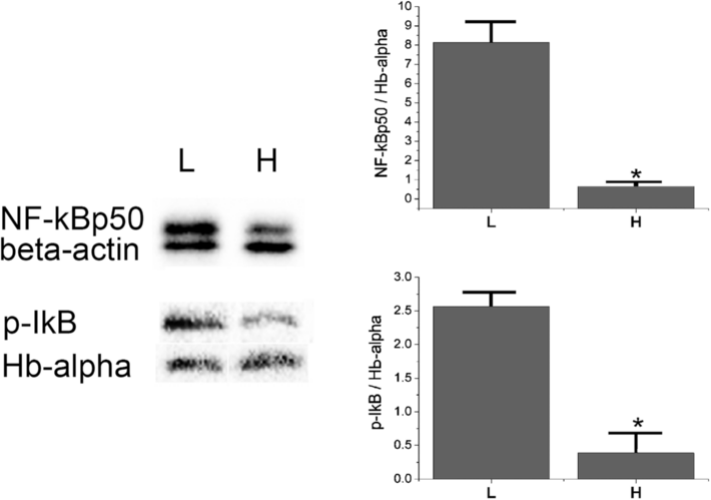
**Western blotting assay for NF-κB p50, phosphorylated-IκB in the lesion segment and far-away hematoma segment of the spinal cord at 3 days post injury. (A)** Representative picture of immunoblots for NF-κB p50, β-actin, phosphorylated-IκB (p-IκB), and hematoglobulin (Hb)-α, respectively. **(B)** Bar graph of ratio of the blot pixels for NF-κB p50 and p-IκB calibrated by Hb-α in lesion and hematoma segments, respectively (*n* = 4 for each group, *P <0.05). H, hematoma; L, lesion site.

In order to compare the inflammatory signal downstream TLR4, we detected the NF-κB p50, phosphorylated inhibitor of kappa B (p-IκB) protein levels of the spinal segment of lesion site and the segment of hematoma at 3 days post injury, based on hemoglobin-α level which was used to represent red blood cells. Repetitive western blotting assay showed that ratio of NF-κB p50/hemoglobin-αlevel was higher in the lesion segment than that in the hematoma segment. The ratio of p-IκB /hemoglobin-α was consistent, statistical analysis showed the differences were both significant (Figure 8).

### Blood-spinal cord barrier compromised

**Figure 9:**
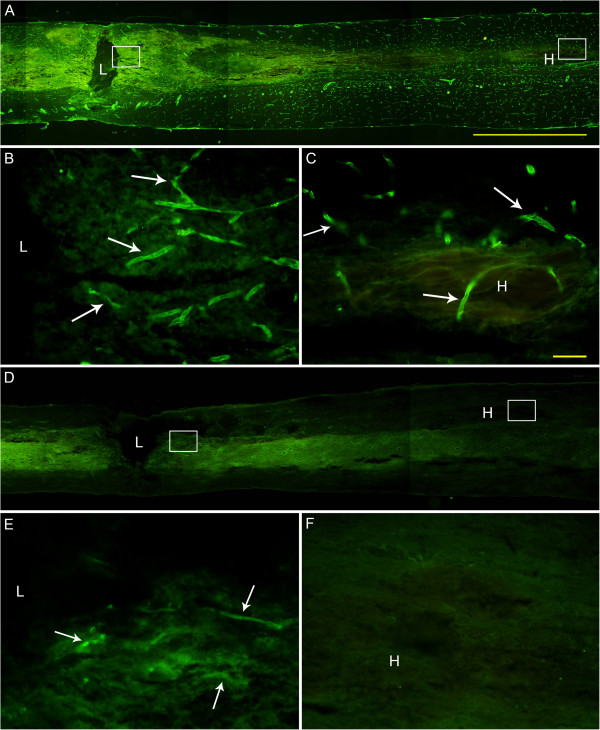
**Microvascular and capillary integrity of the spinal cord in the lesion site and the far-away hematoma. (A)** Representative picture of the longitudinal spinal cord section labeled for RECA without ferric-chloride perfusion at 6 h post injury. **(B)** and **(C)** are amplified pictures of the boxes in lesion site and hematoma, respectively, showing that small blood vessels were there in the lesion site and in hematoma as well. **(D)** Representative picture of the longitudinal spinal cord section labeled for RECA after tannic acid and ferric-chloride perfusion, showing nearly no RECA immunoreactivity. **(E)** and **(F)** are amplified pictures of the boxes in lesion site and hematoma, respectively, showing that RECA immunolabeling only in the lesion site. Bar = 2 mm in (A) and (D), 50 μm in (B), (C), (E) and (F).

Immunohistochemistry for RECA showed that there were blood vessels in lesion site and the far-away hematoma at 6 h post injury. However, spinal cord of animals that were perfused with tannic acid-ferric chloride before sacrifice, RECA positive labeling could only be found in the lesion area, but not in the far-away hematoma, indicated the integrity of blood-vessels in the area of hematoma (Figure 9).

**Figure 10:**
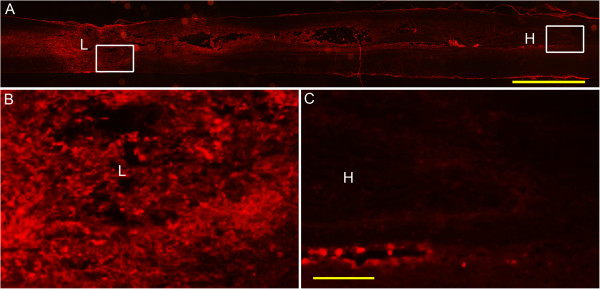
**Representative picture of immunohistochemistry for rat immunoglobulin G (IgG) in the spinal cord at 3 days post injury. (A)** IgG immunoreactive product is obvious in the lesion area (left box), while in the far-away hematoma (right box), there is little immunolabeling for IgG. **(B)** and **(C)** are higher magnification of the boxes in the areas adjacent to the lesion site and to the hematoma, respectively. Bar = 2 mm in (A), 200 μm in (B) and (C).

Shown by immunohistochemistry, rat IgG immunoreactive labeling could be seen in the lesion site and around area of the injured spinal cord at 3 days after compression. However, there was very little IgG immunoreactivity in the far-away hematoma (Figure 10).

## Discussion

A previous study showed in the SCI model that transection injury caused vascular event in the spinal cord distal to the transection site, and exhibited a more pronounced vascular disruption than at comparable sites proximal to the injury [21]. However, we showed in the compressive SCI rat model that the BSCB disruption was less severe in the distal hematoma than in the lesion site. Compared to the ICH model made by blood injection [10,11,22], this far-away hematoma in the spinal cord provides a condition *in vivo* in a natural way that blood component contacts the parenchyma of the CNS. In this model, the innate immune reaction induced by blood could be easily observed.

In this case of far-away hematoma, the profiles of TLR4 response and microglia/macrophage activation were found distinct from that in the lesion center. As described in the results session, although the far-away hematoma appeared at 6 h post injury (it could be even earlier in fact), TLR4 upregulation and microglia/macrophage activation in the far-away hematoma were late and weak, compared to that in the epicenter of the lesion. Generally, at 3 days post injury, TLR4 expression was increased to peak in the epicenter, but TLR4 expression was in low level and microglia/macrophage remained resting in the hematoma area at the same time point. Even later at 14 days post injury, TLR4 and microglia/macrophage activity in the hematoma was not as strong as that in the epicenter at 3 days post injury.

To explain the difference of the innate immune reaction between these blood-comprised foci, one can easily considered that primary injury in the lesion site produced abundant necrotic cells and cell debris, the endogenous DAMP that could activate TLR4; while the area around the far-away hematoma was relatively clear. In addition, heat shock proteins (HSPs) released from the damaged neurons activated microglia [23] and triggered the expression of TLR4 on the surface of microglia/macrophage [24]. In the injured spinal cord, high-mobility group box-1 protein and its receptors including TLR4 were increased at early stage [25]. Necrotic neurons were found to activate microglia via TLR-MyD88 pathway which in turn mediated enhanced neurotoxicity by activated microglia through upregulation of the expression and activity of glutaminase [26]. Based on such environment, the hemorrhage in the lesion site may exacerbate early upregulation of TLR4 in the microglia/macrophage, and initiate pro-inflammation cascade. The hematoma which was far away (around 1.5 cm) from the lesion site, however, containing mainly red blood cells and perhaps a little serum, caused little tissue damage, as shown by H-E staining. This relatively intact tissue environment attributed to the late and weak responses of TLR4 and microglia/macrophage to blood.

Another environmental factor which led to the difference could be the BSCB states in the lesion site and the hematoma area. BSCB compromise could be found in different SCI models by observation of blood-borne protein extravasation. In contusive injury models, BSCB disruption depended on the severity of the injury and days after injury. By 7 days post injury, despite resolution of the initial hemorrhage, there remained scattered evidence for protein extravasation at the injured site and at sites along the axis of the cord [27]. In the impactor-induced spinal cord contusion, such plasma protein extravasated in segments of the spinal cord located away from the compressed part [28]. Here in the present study, BSCB compromise caused by compression mainly restricted in the lesion site, indicated by IgG immunoreactive product. Through the broken BSCB, plasma protein such as albumin extravasated into the parenchyma and induced innate immune reaction mediated by microglia and other neural cells [29].

More importantly, broken capillaries mean hemorrhage and ischemia in the lesion area. A prominent inflammatory response occurs following both ischemic and hemorrhagic stroke, thereby exacerbating secondary injury [30,31]. This study showed clearly with the experiment of carbon powder injection that (at least part of) the blood in the far-away hematoma originated from the lesion site. The hemi-transection aside of the lesion area impeded the occurrence of hematoma far away from the lesion site, which further indicated the identical origination of the hematoma. Such blood origination implied that most local blood vessels and capillaries remained intact in the area far away from the lesion site of the spinal cord. This was proven by the tannic acid-ferric chloride perfusion experiment. As tannic acid-ferric chloride perfusion covered the integrate blood vessels, the endothelial cells could not be labeled by RECA immunohistochemistry; in turn, the RECA labeled were broken capillaries. Without tannic acid-ferric chloride perfusion, RECA immunolabeling showed capillaries both in lesion site and the hematoma, but RECA labeling was seen only in the lesion area after tannic-ferric chloride perfusion, indicating that capillaries, namely BSCB was compromised there. To the contrast, BSCB remained relatively intact in the area adjacent to hematoma, therefore little ischemia or hypoxia occurred this area. Subsequently, the innate immune reaction was weak and late though blood component directly contacted the parenchyma.

Noticeably, the profile of TLR4 and microglia/macrophage responding to hemorrhage in the spinal cord seems different from that in the brain. First, there was nearly no hematoma away from the hemorrhagic or injury center in those ICH models. The far-away hematoma in this SCI model may be due to the spinal cord structure which restricts the hemorrhage to the white matter of the dorsal columns [1]; while the cerebral hemorrhage has little expansion space within the brain parenchyma, so the blood usually stays in the original BBB disruption area. Second, the blood injected to the brain parenchyma seemed comparable to the hematoma away from the epicenter in the spinal cord, but the injected blood in the brain induced TLR4-NF-κB signal activated from 1 day after hemorrhage, and peaked at 3 days [10-12,32], a profile similar to that of epicenter of SCI model. It appears controversial for the blood component to induce TLR4/microglia responses at early phase in the CNS. However, the blood injection was somehow apt to compromise the BBB indicating by brain water content and/or Evans blue extravasation [12]. Even in the experimental subarachnoid cerebral hemorrhage model made by injection of blood into the prechiasmatic cistern, BBB breakdown was found 48 h post injection [22]. Therefore, the BBB compromise at early phase may enhance TLR4 involved innate immune responses in the blood injection models, which is different from the situation of the far-away hematoma in the present study.

It has been well studied that TLR4 activation leads to extensive neuron death *in vitro* that depends on the presence of microglia [33]. Further study demonstrated that HSP60-induced neuronal injury displays characteristics of apoptosis and is dependent on a functional TLR4-MyD88 [24]. In the present study, apoptosis was detected by immunohistochemistry for caspase-3, the key enzyme of the apoptosis progress [34-36], in the epicenter of the lesion site and the area adjacent to the far-away hematoma at different time points which coincide with that of TLR4/microglia activity. According to the result of western blotting assay, the level of p-IκB was higher, namely more NF-κB was free to translocate into the nuclear [37,38], at 3 days post injury in the lesion site than in the hematoma segment based on the similar hematoglobin level. These data are consistent with the previous reports about microglial TLR4/NF-κB signal induced subsequent neural cell apoptosis [24,39].

In addition to the late and weak upregulation, TLR4 expression and microglia/macrophage activation in the far-away hematoma were closely associated with phagocytosis, as indicated by morphological observation that TLR4 immunoreactive product was located within the microglia/macrophages. While in the lesion site, such phagocytotic microglia/macrophages with TLR4 immunoreactivity did not appear so obvious. As an innate immune receptor, TLR4 plays roles in clearance of DAMPs such as A-beta peptides [23,40], and microglia/macrophage also delete the cell debris by phagocytosis [41]. Meanwhile, cavitation occurred in the site of the far-away hematoma as observed at 14 days post injury. Therefore, TLR4 and microglia/macrophage phagocytosis in the present study may link the clearance of the blood and the cavitation in the spinal cord, particularly in the hematoma. Since the cavity formation in the spinal cord is associated with protection to the spared cord and recovery from the injury [42], these TLR4 and microglia/macrophage responses should benefit recovery from the spinal cord injury.

The different profiles of TLR4 and microglia/macrophage responses to the intraparenchymal hemorrhage further reflect the complexity of SCI. Owing to its complexity, a spinal cord injury is unlikely to be cured by a single therapy [17]. Even TLR4 is taken as the therapeutic target, the proper time window of treatment as well as the innate immune environment of the spinal cord are the prerequisites for a success therapy.

TLRs have been implicated to play important roles in both tissue surveying and repair, and have been concerned as potential therapeutic targets in CNS inflammation [3], while TLRs can exert either beneficial or detrimental effects on the CNS, which probably depend on the context of tissue homoeostasis or pathology. Therefore, any potential therapeutic manipulation of TLRs will require an understanding of the signals governing specific CNS disorders to achieve tailored therapy [43]. TLR4 deficiency has been proved by Kilic and colleagues to be protective to neurons against ischemia and axotomy [44], but Kigerl and colleagues found that TLR2 and TLR4 deficiency may impair the locomotor function recovery from SCI [45]. These controversial conclusions might be due to the differences between the injury models, while the immune microenvironment should be more important impact factor that could not be excluded.

As this study was a primary exploration to the hemorrhage-induced innate immune reaction in the spinal cord, all these data provided here may be insufficient to illustrate precisely the relationship between TLR4/microglia responses and the amount of the blood. To answer better how blood component and/or other factor(s) mediate the innate immune response in SCI, more experiments need to be performed next step, for instance, TLR4/microglia responses to blood *in vitro* and observation to the TLR4/microglia responses at SCI model with repaired BSCB.

## Conclusion

As far as our knowledge, this is the first evidence about hemorrhage-induced TLR4 expression and microglia/macrophage activation in the spinal cord of the rats. With far-away hematoma originated from the lesion site hemorrhage in the spinal parenchyma, we showed distinct profiles of TLR4 and microglia/macrophage activation due to different innate immune environments. These data suggest that hemorrhage in the spinal cord induce the innate immune reaction involving TLR4 and microglia/macrophage activities but require other conditions including tissue/cell damage and BSCB disruption. This study also indicates that hemorrhage in the spinal cord may be different from that in the brain, and the complexity and the trait of SCI should be considered in the therapeutic strategy targeting the innate immune responses.

## Abbreviations

BBB: Blood–brain barrier; BSCB: Blood-spinal cord barrier; CNS: Central nervous system; DAMP: Damage associated molecule pattern; H-E: Hematoxylin and eosin; HSP: Heat shock protein; ICH: Intracerebral hemorrhage; MyD88: Myeloid differentiation factor 88; NF-κB: Nuclear factor-κB; p-IκB: Phosphor-inhibitor of NF-κB; PAMP: Pathogen associated molecule pattern; RECA: Reactive endothelial cell antigen; SCI: Spinal cord injury; TLR: Toll-like receptor

## Competing interests

The authors declare that they have no competing interests.

## Authors’ contributions

ZYK made all the animal models, performed most immunohistochemistry and H-E staining; LJT, PZW, and FH performed western blotting assay and analysis; YAH, PC, and LL participated in the animals’ care, tissue preparation, and sectioning; JG gave important directions to the model design and morphological analysis; KF was involved in the study design, data analysis, and manuscript writing. All authors read and approved the final manuscript.
